# Construction of g-C_3_N_4_-mNb_2_O_5_ Composites with Enhanced Visible Light Photocatalytic Activity

**DOI:** 10.3390/nano8060427

**Published:** 2018-06-12

**Authors:** Meiyin Wang, Hui Wang, Yuanhang Ren, Cheng Wang, Zhewei Weng, Bin Yue, Heyong He

**Affiliations:** Department of Chemistry and Shanghai Key Laboratory of Molecular Catalysis and Innovative Materials, Collaborative Innovation Center of Chemistry for Energy Materials, Fudan University, Shanghai 200433, China; 14110220018@fudan.edu.cn (M.W.); 17210220053@fudan.edu.cn (H.W.); YuanhangRen@fudan.edu.cn (Y.R.); 15110220022@fudan.edu.cn (C.W.); 16210220004@fudan.edu.cn (Z.W)

**Keywords:** photocatalytic degradation, g-C_3_N_4_, mesoporous Nb_2_O_5_, organic pollutant

## Abstract

A series of composites consisting of g-C_3_N_4_ sheet and mesoporous Nb_2_O_5_ (mNb_2_O_5_) microsphere were fabricated by in situ hydrolysis deposition of NbCl_5_ onto g-C_3_N_4_ sheet followed by solvothermal treatment. The samples were characterized using powder X-ray diffraction (XRD), Fourier transform infrared spectroscopy (FT-IR), transmission electron microscopy (TEM), N_2_ adsorption-desorption, X-ray photoelectron spectroscopy (XPS), UV-vis diffuse reflectance spectroscopy (DRS) and photoluminescence spectroscopy (PL). The photocatalytic activity of the composites was studied by degradation of rhodamine B (RhB) and tetracycline hydrochloride (TC-HCl) in aqueous solution under visible light irradiation (*λ* > 420 nm). Compared with g-C_3_N_4_ and mNb_2_O_5_, g-C_3_N_4_-mNb_2_O_5_ composites have higher photocatalytic activity due to synergistic effect between g-C_3_N_4_ and mNb_2_O_5_. Among these composites, 4% g-C_3_N_4_-mNb_2_O_5_ has the highest efficiency and good recyclability for degradation of both RhB and TC-HCl.

## 1. Introduction

Organic dyes and antibiotics are two types of important products which are widely used in textile and pharmaceutical industries, respectively. The direct discharge of these chemical compounds along with sewage to environment would be seriously harmful to ecosystem and human health. It is, therefore, desirable to explore efficient ways to remove them from water [1,2,3]. In the past decades, the methods of biological treatment, physical adsorption and chemical transformation have been employed in removal of the organic pollutants [1,4,5,6,7]. Among them, much attention has been focused on photocatalytic degradation of organic pollutants over semiconductors such as TiO_2_ [7,8], WO_3_ [9], ZnO [10], MoS_2_ [11], etc. due to their ability to oxidize organics through a redox process with low cost. Nevertheless, the majority of the above semiconductors still suffer from some problems such as limited absorption of visible light, difficult recycling of the catalyst, rapid recombination of photogenerated electrons, holes pairs, etc. n-Type transition metal oxide Nb_2_O_5_ attracts much interest in photocatalytic reactions due to its high chemical stability, water tolerance, and nontoxicity, although the light absorption of Nb_2_O_5_ with a wide band gap of ~3.4 eV limits its application only in the UV region [12,13,14]. Therefore, many efforts have been made to improve the absorption ability of Nb_2_O_5_ in the visible region, such as combination with other semiconductor material [15], ion doping [16] and deposition of noble metal [17].

Recently, graphitic carbon nitride (g-C_3_N_4_) has been widely used in photocatalytic reactions such as degradation of pollutants, hydrogen generation and selective oxidation of alcohols under visible light due to its excellent chemical/thermal stability, small band gap of 2.7 eV and low cost [18,19,20]. However, pure g-C_3_N_4_ shows low photocatalytic efficiency owing to fast recombination of photogenerated electrons and holes and small specific surface area [21,22]. It was reported that the composites of g-C_3_N_4_ and other semiconductors, such as TiO_2_ [23,24,25], WO_3_ [26], MoS_2_ [27], and ZnO [28], could produce a certain number of heterojunction sites which is beneficial to promote electron-hole separation and restrain the recombination efficiently. In addition, some efforts have been paid to the Nb_2_O_5_/g-C_3_N_4_ composite which exhibits photocatalytic activity in degradation of tetracycline hydrochloride (TC-HCl) [29], methylene blue (MB) and rhodamine B (RhB) under UV and visible light [30], and production of H_2_ under visible light [31]. However, it is still a challenge to prepare novel Nb_2_O_5_/g-C_3_N_4_ composites with strong interaction and high dispersion between mesoporous Nb_2_O_5_ microsphere and g-C_3_N_4_ layer which could be used as efficient photocatalysts.

Herein, a series of g-C_3_N_4_-mNb_2_O_5_ composites, prepared by in situ hydrolysis deposition of NbCl_5_ onto g-C_3_N_4_ sheet followed by solvothermal treatment, have been used as photocatalysts in degradation of RhB and TC-HCl and characterized in details. The results indicate that 4% g-C_3_N_4_-mNb_2_O_5_ exhibits low photoluminescence (PL) intensity and narrow band gap which account for its high catalytic activity.

## 2. Materials and Methods

### 2.1. Materials

Niobium chloride (NbCl_5_) was purchased from Strem Chemicals, Inc (Newburyport, MA, USA). Melamine (99%), absolute ethanol (analytical grade) and tetracycline hydrochloride (TC-HCl, 96%) were purchased from Shanghai Aladdin Bio-Chem Technology Co., Ltd. (Shanghai, China). Rhodamine B (RhB) was purchased from Sinopharm Chemical Reagent Co., Ltd. (Shanghai, China). Pluronic 123 (PEG-PPG-PEG, P123) was purchased from Sigma-Aldrich, Co. (ST. Louis, MO, USA). All the reagents were used without further purification.

### 2.2. Preparation

The bulk g-C_3_N_4_ was prepared by heating 2.50 g of melamine in an alumina crucible with a cover in air at 550 °C with ramp rate of 2 °C/min and maintained at 550 °C for 4 h [32]. After being cooled down to room temperature, the obtained product was ground into powder.

The g-C_3_N_4_-mNb_2_O_5_ composites were prepared through in situ hydrolysis of NbCl_5_ onto g-C_3_N_4_ and then solvothermal treatment. Typically, 0.99 g of P123 was added into 20 g of absolute ethanol with vigorous stirring until complete dissolution of P123. Bulk g-C_3_N_4_ powder was dispersed into absolute ethanol and the suspension was sonicated for 1 h. Then, 1.49 g of NbCl_5_ was added into a pear-shaped flask under N_2_ atmosphere in a glovebox. The solution of P123 and the suspension of g-C_3_N_4_ were added and the mixture was stirred for 30 min. After that, 0.50 mL of distilled water was pumped into the flask with a rate of 0.0167 mL/min under stirring. After stirring for another 30 min, the mixture was transferred into a 40 mL Teflon-lined stainless steel autoclave and heated at 180 °C for 24 h. After being cooled down to room temperature, the precipitate was obtained by filtration and washing by ethanol for four times. The solid was dried at 30 °C overnight in a drying oven under vacuum and then transferred to a tube furnace to be calcined at 400 °C for 3 h with a rate of 2 °C/min. The g-C_3_N_4_-mNb_2_O_5_ composites prepared with the g-C_3_N_4_ weight ratios of 1%, 4%, 10%, 20% and 50% were denoted as 1% g-C_3_N_4_-mNb_2_O_5_, 4% g-C_3_N_4_-mNb_2_O_5_, 10% g-C_3_N_4_-mNb_2_O_5_, 20% g-C_3_N_4_-mNb_2_O_5_, 50% g-C_3_N_4_-mNb_2_O_5_, respectively. The pure mNb_2_O_5_ was prepared by the same procedure without adding g-C_3_N_4_. In addition, the corresponding mechanic mixture of g-C_3_N_4_ and mNb_2_O_5_ with the same weight ratio of 4% g-C_3_N_4_-mNb_2_O_5_ was prepared by simple grinding and was denoted as 4% g-C_3_N_4_/mNb_2_O_5_.

### 2.3. Characterization

Powder X-ray diffraction (XRD) patterns were carried out on a Bruker D8 Advance diffractometer (Karlsruhe, Germany) with Cu K*α* radiation (*λ* = 0.15418 nm) operated at 40 kV and 40 mA in the 2*θ* range of 5–70°. The Fourier transform infrared (FT-IR) spectra were recorded on a Thermo Fisher Nicolet iS10 instrument (Waltham, MA, USA) with KBr pellet from 4000 to 400 cm^−1^. X-ray photoelectron spectroscopy (XPS) was measured by a Perkin Elmer PHI 5000C spectroscope (Waltham, MA, USA). The spectra were recorded with Mg K*α* line as the excitation source (*hν* = 1253.6 eV) at 14 kV and 20 mA. UV-vis diffuse reflectance spectroscopy (DRS) was performed on a Perkin Elmer Lambda 650 spectrophotometer (Waltham, MA, USA) using BaSO_4_ as reference. The transmission electron microscopic (TEM) images were obtained using a FEI Tecnai G^2^ F20 S-Twin field emission transmission electron microscope (Hillsboro, Oregon, USA) with an accelerating voltage of 200 kV and a JEOL JEM-2011 transmission electron microscope with an accelerating voltage of 200 kV. The samples were prepared by dropping the ethanol suspension of samples onto the copper grid. The N_2_ adsorption-desorption measurements were carried out at 77.3 K on a Micromeritics Tristar II 3020 analytical system. The specific surface area was calculated by the Brunauer-Emmett-Teller (BET) method. Pore size distributions were obtained from analysis of the desorption branch of the isotherms using the Barrett-Joyner-Halenda (BJH) model. Photoluminescence (PL) spectra were obtained on an Edinburgh Instruments FLS-980 spectrometer (Edinburgh, UK) with the excitation wavelength of 300 nm.

### 2.4. Photocatalytic Experiments

The photocatalytic activity of the as-prepared catalysts was performed by photocatalytic degradation of 15 mg/L RhB aqueous solution and 40 mg/L TC-HCl aqueous solution under visible light. The photocatalytic degradation tests were carried out in an instrument (CEL-HXF300, Beijing China Education Au-light Co., Ltd., Beijing, China) with a cylindrical glass reactor and a condensate water circulation equipment (Figure S1). The visible light was provided by a Xe lamp (14 V, 15 A) with a 420 nm cut-off filter (Figure S2). In a typical photocatalytic degradation of RhB or TC-HCl experiment, 30 mg of catalyst was added into 70 mL of RhB aqueous solution or 100 mL of TC-HCl aqueous solution. The suspension was firstly stirred in dark at room temperature to reach adsorption/desorption equilibrium. Then, the reaction was irradiated under visible light (*λ* > 420 nm) and the solution was vigorously stirred with air bubbling. During the process, 3 mL of suspension was taken at intervals of 20 min (for RhB) or intervals of 5 min (for TC-HCl). All suspensions were centrifuged to remove the catalyst and the concentration of the RhB and TC-HCl were analyzed by UV-vis spectrophotometer with the scanning ranges of 300–700 nm for RhB and 200–600 nm for TC-HCl. To be reused for next reaction cycle, the separated catalyst by centrifugation was washed by water and ethanol three times and then dried at 30 °C in a drying oven under vacuum.

## 3. Results and Discussion

### 3.1. Characterization of the Catalysts

#### 3.1.1. XRD

**Figure 1:**
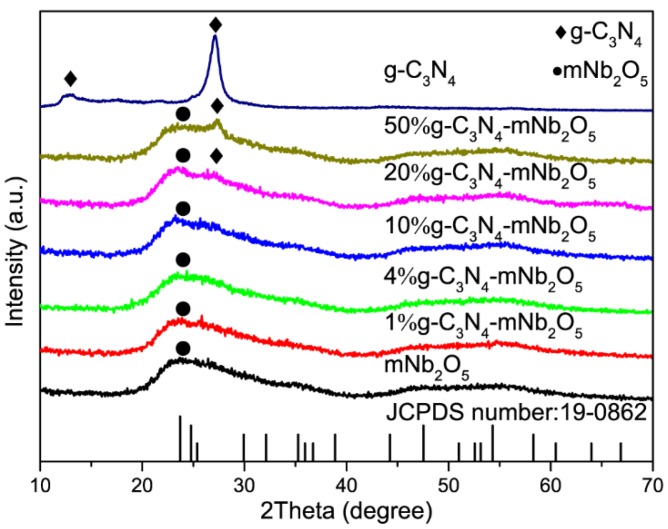
XRD patterns for mNb_2_O_5_, g-C_3_N_4_ and g-C_3_N_4_-mNb_2_O_5_ composites with different contents of g-C_3_N_4_.

XRD was employed to analyze mNb_2_O_5_, g-C_3_N_4_ and g-C_3_N_4_-mNb_2_O_5_ composites with different contents of g-C_3_N_4_ and the results are shown in Figure 1. For mNb_2_O_5_, the broad diffraction peak around 23.7° corresponds to partial crystallized Nb_2_O_5_ (JCPDS number: 19-0862), which results from the relatively low calcination temperature of 400 °C. The main diffractions of g-C_3_N_4_ are at 12.8° and 27.1°, which are assigned to (100) plane due to in-plane tris-s-triazine frameworks and (002) plane due to interlayer stacking of aromatic systems, respectively. The latter reflects interlayer stacking of aromatic systems and the calculated interlayer distance is 0.338 nm [33,34,35]. In the composites, the diffractions of mNb_2_O_5_ are retained and the characteristic (002) diffraction of g-C_3_N_4_ is observed when the content of g-C_3_N_4_ increases to 20% and 50%.

#### 3.1.2. FT-IR

**Figure 2:**
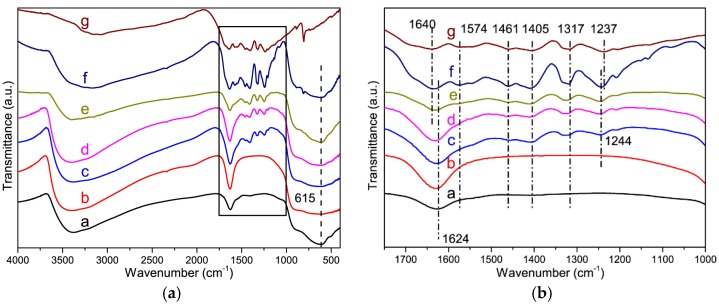
(**a**) FT-IR spectra of mNb_2_O_5_, g-C_3_N_4_ and g-C_3_N_4_-mNb_2_O_5_ composites with different contents of g-C_3_N_4_. (a, mNb_2_O_5_; b, 1% g-C_3_N_4_-mNb_2_O_5_; c, 4% g-C_3_N_4_-mNb_2_O_5_; d, 10% g-C_3_N_4_-mNb_2_O_5_; e, 20% g-C_3_N_4_-mNb_2_O_5_; f, 50% g-C_3_N_4_-mNb_2_O_5_; g, g-C_3_N_4_); and (**b**) enlarged FT-IR spectra corresponding to rectangle region from (**a**).

Figure 2 shows the FT-IR spectra of mNb_2_O_5_, g-C_3_N_4_ and g-C_3_N_4_-mNb_2_O_5_ composites. For the mNb_2_O_5_ sample, a broad band around 3382 cm^−1^ is attributed to vibration of O-H of the water molecules adsorbed on the sample [27,30]. The broad band at 615 cm^−1^ is ascribed to Nb–O–Nb angular vibration [36]. As for g-C_3_N_4_, the broad band ranging from 3000 to 3500 cm^−1^ is due to the stretching vibration of N–H groups in g-C_3_N_4_ and O–H of adsorbed water [25]. Other characteristic bands arisen from the typical stretching modes of C=N and C–N in heterocycles of g-C_3_N_4_ are at 1237, 1317, 1405, 1461, 1574 and 1640 cm^−1^. Additionally, the band at 803 cm^−1^ of g-C_3_N_4_ is assigned to the breathing mode of the triazine units [33,34]. For g-C_3_N_4_-mNb_2_O_5_ composites, all the characteristic bands of mNb_2_O_5_ and g-C_3_N_4_ exist except 1% g-C_3_N_4_-mNb_2_O_5_ due to small amount of g-C_3_N_4_, which confirm the existence of two components in the composites. With the increasing of g-C_3_N_4_, the band at 3382 cm^−1^ of mNb_2_O_5_ slightly shifts to smaller wavenumber for g-C_3_N_4_-mNb_2_O_5_ composites, which reveals the vibration of N-H. Moreover, the band of g-C_3_N_4_ at 1237 cm^−1^ slightly shifts to 1244 cm^−1^ for all g-C_3_N_4_-mNb_2_O_5_ composites except 1% g-C_3_N_4_-mNb_2_O_5_ (Figure 2b), revealing the interaction between g-C_3_N_4_ and mNb_2_O_5_.

#### 3.1.3. TEM

**Figure 3:**
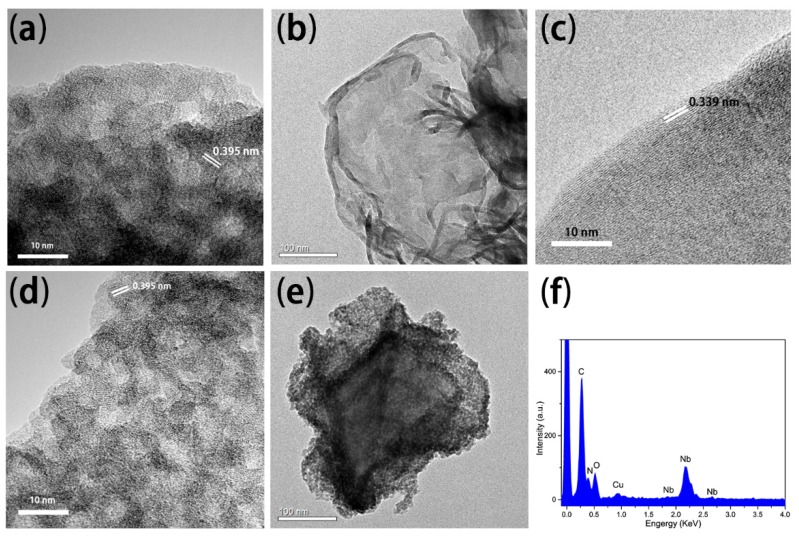
TEM images of: (**a**) mNb_2_O_5_; (**b**,**c**) g-C_3_N_4_; and (**d**,**e**) 4% g-C_3_N_4_-mNb_2_O_5_; (**f**) EDS analysis of 4% g-C_3_N_4_-mNb_2_O_5_ corresponding to (**e**).

The prepared samples were investigated by field emission transmission electron microscope. Figure 3 shows the TEM images of g-C_3_N_4_, mNb_2_O_5_ and 4% g-C_3_N_4_-mNb_2_O_5_. The mNb_2_O_5_ sample is mesoporous materials with pore size of 3–5 nm and its lattice fringe spacing is 0.395 nm (Figure 3a). Figure 3b shows that g-C_3_N_4_ is veil-like with lamellar structure. In Figure 3c, we could measure that the interlayer distance g-C_3_N_4_ is 0.339 nm, which is in accordance with XRD result. For the 4% g-C_3_N_4_-mNb_2_O_5_ sample, it can be observed that mNb_2_O_5_ microspheres are deposited on the surface of g-C_3_N_4_ sheet in Figure 3d,e. Moreover, the corresponding EDS of Figure 3e indicates that the sample consists of C, N, O and Nb elements (Figure 3f). The relevant element contents are shown in Table S1.

#### 3.1.4. N_2_ Adsorption-Desorption

**Figure 4:**
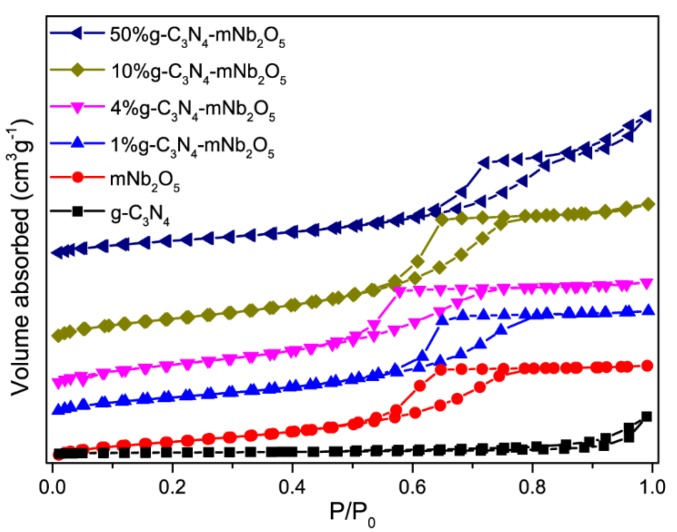
Nitrogen adsorption–desorption isotherms of g-C_3_N_4_, mNb_2_O_5_, 1% g-C_3_N_4_-mNb_2_O_5_, 4% g-C_3_N_4_-mNb_2_O_5_, 10% g-C_3_N_4_-mNb_2_O_5_ and 50% g-C_3_N_4_-mNb_2_O_5_.

Figure 4 shows the nitrogen adsorption-desorption isotherms of g-C_3_N_4_, mNb_2_O_5_, 1% g-C_3_N_4_-mNb_2_O_5_, 4% g-C_3_N_4_-mNb_2_O_5_, 10% g-C_3_N_4_-mNb_2_O_5_ and 50% g-C_3_N_4_-mNb_2_O_5_. The isotherm of g-C_3_N_4_ belongs to the type IV isotherm with the H4 type hysteresis loop. All isotherms of mNb_2_O_5_, 1% g-C_3_N_4_-mNb_2_O_5_, 4% g-C_3_N_4_-mNb_2_O_5_, 10% g-C_3_N_4_-mNb_2_O_5_ and 50% g-C_3_N_4_-mNb_2_O_5_ are ascribed to the type V isotherm with the H3 type hysteresis loop, indicating the existence of mesoporous structure. The BET specific surface areas of g-C_3_N_4_ and mNb_2_O_5_ are 11.2 and 138 m^2^/g, respectively. The surface area of the composites follows the sequence: 4% g-C_3_N_4_-mNb_2_O_5_ (190 m^2^/g) > 10% g-C_3_N_4_-mNb_2_O_5_ (182 m^2^/g) > 1% g-C_3_N_4_-mNb_2_O_5_ (152 m^2^/g) > 50% g-C_3_N_4_-mNb_2_O_5_ (136 m^2^/g). Among them, 4% g-C_3_N_4_-mNb_2_O_5_ exhibits the largest surface area, which is 1.4 times larger than that of mNb_2_O_5_ and 17 times larger than that of g-C_3_N_4_. It may originate from that, after ultrasonication of g-C_3_N_4_, the interlayer distance of g-C_3_N_4_ increases and the addition of g-C_3_N_4_ leads to partial disaggregation of mNb_2_O_5_ [37,38]. When the content of g-C_3_N_4_ is 50%, high content of g-C_3_N_4_ in the composites results in g-C_3_N_4_ stacking together, thus 50% g-C_3_N_4_-mNb_2_O_5_ sample shows almost the same specific surface as mNb_2_O_5_.

#### 3.1.5. UV-vis DRS

**Figure 5:**
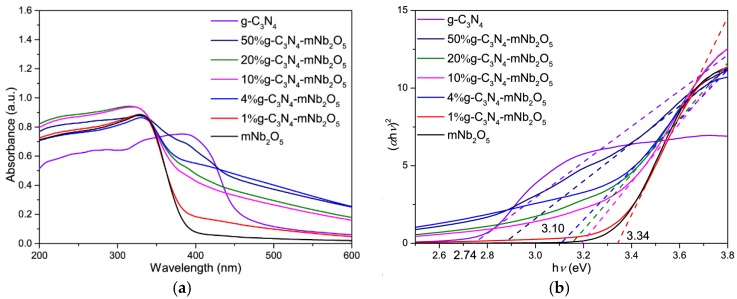
(**a**) UV-vis diffuse reflectance spectra; and (**b**) plots of the (*α*h*υ*)^2^ vs. (h*υ*) of mNb_2_O_5_, g-C_3_N_4_, and g-C_3_N_4_-mNb_2_O_5_ composites with different content of g-C_3_N_4_.

UV-vis diffuse reflectance spectroscopy (DRS) was performed to estimate the band gap of the catalysts, which is important to determine if the catalysts can be excited in the visible-light region [39]. Figure 5a shows the UV-vis diffuse reflectance spectra of mNb_2_O_5_, g-C_3_N_4_ and g-C_3_N_4_-mNb_2_O_5_ composites with different content of g-C_3_N_4_. Due to the high band energy value of mNb_2_O_5_ (3.34 eV), mNb_2_O_5_ only absorbs ultraviolet light with its fundamental absorption edge near 371 nm. However, g-C_3_N_4_ possesses a broad peak in the visible region with an absorption edge at ca. 458 nm. For g-C_3_N_4_-mNb_2_O_5_ composites, the absorption edge exhibits an obvious red shift relative to pristine mNb_2_O_5_, indicating the composites could absorb more visible light than mNb_2_O_5_. The band gap energy (*Eg*) of the samples was determined by UV-vis DRS with the Tauc model according to the following equation:

*α*h*υ* = A(h*υ* − *E_g_*)^n/2^ (1)

where *α*, h, *υ* and A corresponds to absorption coefficient, Planck constant, light frequency and a constant, respectively, and the constant n depends on whether the transition is direct (n = 1) or indirect (n = 4) [40]. For both g-C_3_N_4_ and mNb_2_O_5_, the values of n are 1 [41,42]. Thus, the band gap values of 1% g-C_3_N_4_-mNb_2_O_5_, 4% g-C_3_N_4_-mNb_2_O_5_, 10% g-C_3_N_4_-mNb_2_O_5_, 20% g-C_3_N_4_-mNb_2_O_5_ and 50% g-C_3_N_4_-mNb_2_O_5_ are estimated as 3.34, 3.10, 3.19, 3.15 and 2.88 eV, respectively (Figure 5b). The narrowed band gaps of 4% g-C_3_N_4_-mNb_2_O_5_, 10% g-C_3_N_4_-mNb_2_O_5_ and 20% g-C_3_N_4_-mNb_2_O_5_ results from the formation of heterostructure between g-C_3_N_4_ and mNb_2_O_5_ [29]. It implies that 4% g-C_3_N_4_-mNb_2_O_5_ can harvest more visible light, which is beneficial to improve the visible-light photocatalytic activity of catalysts. Interestingly, the band gap of 50% g-C_3_N_4_-mNb_2_O_5_ is lower than that of other composites, due to high content of g-C_3_N_4_ in 50% g-C_3_N_4_-mNb_2_O_5_.

#### 3.1.6. XPS

**Figure 6:**
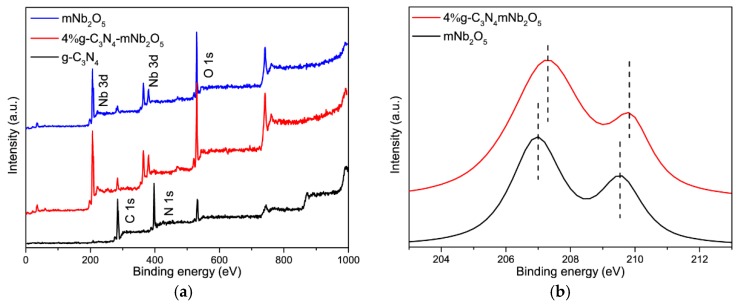
(**a**) XPS survey spectra of g-C_3_N_4_, mNb_2_O_5_ and 4% g-C_3_N_4_-mNb_2_O_5_; and (**b**) Nb 3d spectra for mNb_2_O_5_ and 4% g-C_3_N_4_-mNb_2_O_5_.

XPS was used to characterize the surface chemical composition and elemental valence states of the samples. As shown in Figure 6a, C and N elements are detected from g-C_3_N_4_ and Nb and O elements are detected from mNb_2_O_5_. As for the spectrum of 4% g-C_3_N_4_-mNb_2_O_5_, a very weak N 1s peak is ascribed to small content of g-C_3_N_4_. Figure 6b presents the high resolution XPS spectra of Nb 3d for 4% g-C_3_N_4_-mNb_2_O_5_ and mNb_2_O_5_. For mNb_2_O_5_, the signals of Nb 3d_5/2_ and 3d_3/2_ locate at 207.0 and 209.6 eV, respectively, whereas the signals of Nb 3d_5/2_ and 3d_3/2_ shift slightly (ΔBE ~ 0.4 eV) to a higher binding energy for 4% g-C_3_N_4_-mNb_2_O_5_. The upshift may be attributed to band bending.

#### 3.1.7. PL Spectroscopy

**Figure 7:**
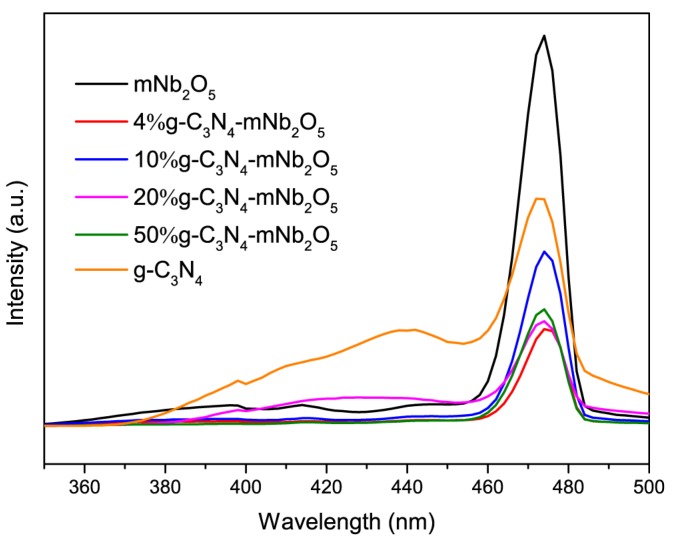
PL spectra of mNb_2_O_5_, 4% g-C_3_N_4_-mNb_2_O_5_, 10% g-C_3_N_4_-mNb_2_O_5_, 20% g-C_3_N_4_-mNb_2_O_5_, 50% g-C_3_N_4_-mNb_2_O_5_ and g-C_3_N_4_.

To test the generation and recombination efficiency of photogenerated electrons and holes in semiconductors, PL spectroscopy is often conducted. The PL spectra of mNb_2_O_5_, 4% g-C_3_N_4_-mNb_2_O_5_, 10% g-C_3_N_4_-mNb_2_O_5_, 20% g-C_3_N_4_-mNb_2_O_5_, 50% g-C_3_N_4_-mNb_2_O_5_ and g-C_3_N_4_ with an excitation wavelength of 300 nm are shown in Figure 7. It can be observed that mNb_2_O_5_ has a strong emission peak at 474 nm and g-C_3_N_4_ has a strong emission peak at 472 nm. Compared to mNb_2_O_5_, the PL emission intensity of g-C_3_N_4_-mNb_2_O_5_ samples is significantly reduced, suggesting that the g-C_3_N_4_-mNb_2_O_5_ composite has a lower recombination rate of photogenerated electrons and holes than mNb_2_O_5_. Among the composites, the 4% g-C_3_N_4_-mNb_2_O_5_ sample shows the lowest emission intensity at the similar emission peak position which means the lowest recombination efficiency of photogenerated electrons and holes [27,43,44]. Combined with the UV-vis DRS analysis result that 4% g-C_3_N_4_-mNb_2_O_5_ has the lowest band gap, 4% g-C_3_N_4_-mNb_2_O_5_ demonstrates best potential in photocatalytic degradation reactions among the catalysts studied in this work.

### 3.2. Visible-Light Photocatalytic Performance and Stability of the Catalysts

The photocatalytic test of mNb_2_O_5_, g-C_3_N_4_ and g-C_3_N_4_-mNb_2_O_5_ composites with different content of g-C_3_N_4_ in degradation of RhB and TC-HCl were carried out under visible light irradiation (*λ* > 420 nm).

#### 3.2.1. Photodegradation of RhB under Visible Light Irradiation

**Figure 8:**
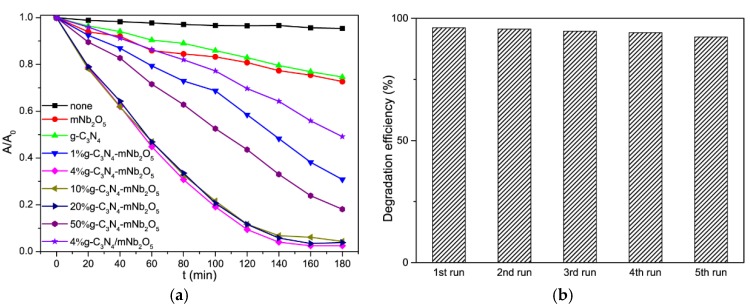
(**a**) Photolysis of RhB and photocatalytic activity over as-prepared photocatalysts for RhB; and (**b**) recyclability for the photodegradation of RhB in the presence of 4% g-C_3_N_4_-mNb_2_O_5_ under visible light irradiation.

The photocatalytic degradation results are shown in Figure 8a. The standard curve of the absorbance intensity (A) vs. concentration (C) of RhB solution is shown in Figure S4a. The RhB suspension was firstly stirred in dark for 2 h at room temperature to reach adsorption/desorption equilibrium (Figure S3a) [45,46]. No evident decrease of RhB concentration is observed in the absence of catalyst. Therefore, the direct photolysis of RhB could be ignored. Pure mNb_2_O_5_ and g-C_3_N_4_ show low photocatalytic activities with only about 27.3% and 25.4% of RhB being degraded after 180 min of irradiation, respectively. The g-C_3_N_4_-mNb_2_O_5_ composites show higher degradation efficiency than mNb_2_O_5_ and g-C_3_N_4_. For the catalysts containing different content of g-C_3_N_4_, the sample 4% g-C_3_N_4_-mNb_2_O_5_ exhibits the highest degradation efficiency for RhB (97.5%) within 180 min, which is 3.6 and 3.9 times higher than that of mNb_2_O_5_ and g-C_3_N_4_, respectively. Furthermore, the corresponding mechanic mixture 4% g-C_3_N_4_/mNb_2_O_5_ shows 51% of degradation efficiency for RhB, which is much smaller than that of 4% g-C_3_N_4_-mNb_2_O_5_ composite. Therefore, the high degradation efficiency of 4% g-C_3_N_4_-mNb_2_O_5_ is ascribed to the formation of heterostructure between g-C_3_N_4_ and mNb_2_O_5_, as found in the UV-vis DRS and PL studies.

In addition to photocatalytic efficiency, stability and recyclability of the catalysts are also important for application of the catalysts. The 4% g-C_3_N_4_-mNb_2_O_5_ sample for photodegradation of RhB was further tested for five cycles. As shown in Figure 8b, after five cycles, the high efficiency for photocatalytic degradation of RhB by 4% g-C_3_N_4_-mNb_2_O_5_ is maintained, demonstrating good reusability and stability of 4% g-C_3_N_4_-mNb_2_O_5_.

#### 3.2.2. Photodegradation of TC-HCl under Visible Light Irradiation

**Figure 9:**
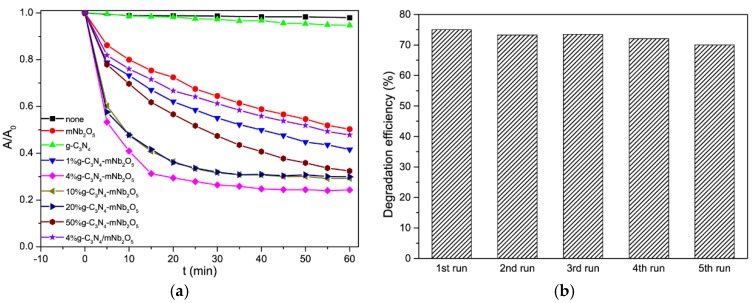
(**a**) Photolysis of TC-HCl and photocatalytic activity over as-prepared photocatalysts for TC-HCl; and (**b**) recyclability for the photodegradation of TC-HCl in the presence of 4% g-C_3_N_4_-mNb_2_O_5_ under visible light irradiation.

The photocatalytic degradation of TC-HCl results are shown in Figure 9a. The standard curve of the absorbance intensity (A) vs. concentration (C) of TC-HCl solution can be seen in Figure S4b. The TC-HCl suspension was firstly stirred in dark for 1 h at room temperature to reach adsorption/desorption equilibrium (Figure S3b) [45,46]. No evident decrease of TC-HCl concentration is observed in the absence of catalyst. The pure mNb_2_O_5_ and g-C_3_N_4_ show low photocatalytic efficiency in degradation of TC-HCl with about 49.7% and 5.3% after 60 min of irradiation, respectively. The degradation efficiency of g-C_3_N_4_-mNb_2_O_5_ composites within 60 min could be listed as the following order: 4% g-C_3_N_4_-mNb_2_O_5_ > 10% g-C_3_N_4_-mNb_2_O_5_ ≈ 20% g-C_3_N_4_-mNb_2_O_5_ > 50% g-C_3_N_4_-mNb_2_O_5_ > 1% g-C_3_N_4_-mNb_2_O_5_. All g-C_3_N_4_-mNb_2_O_5_ composites display higher degradation efficiency than that of mNb_2_O_5_ and g-C_3_N_4_. For comparison, the corresponding mechanic mixture 4% g-C_3_N_4_/mNb_2_O_5_ shows its degradation efficiency of 52%. Overall, 4% g-C_3_N_4_-mNb_2_O_5_ is the optimal catalyst, for which the degradation efficiency of TC-HCl in 60 min is 75.7%. After 30-min experimental run, for 4% g-C_3_N_4_-mNb_2_O_5_, 10% g-C_3_N_4_-mNb_2_O_5_ and 20% g-C_3_N_4_-mNb_2_O_5_, degradation reaction of TC-HCl solution has approached the endpoint. Further degradation is hard to proceed so that the degradation rate is slow and the change of absorbance change is not evident. An appropriate content of g-C_3_N_4_ leads to this composite with large surface area, narrow band gap and low PL intensity, which favors the titled photocatalytic reactions [41,44,47].

The stability and recyclability of 4% g-C_3_N_4_-mNb_2_O_5_ catalysts for photocatalytic degradation of TC-HCl were also tested for five cycles. As shown in Figure 9b, after five cycles, photodegradation efficiency of 4% g-C_3_N_4_-mNb_2_O_5_ for TC-HCl is almost unchanged. Thus, 4% g-C_3_N_4_-mNb_2_O_5_ sample could maintain good stability and it is able to be reused in the photodegradation reaction of TC-HCl as well as in photodegradation reaction of RhB.

In other words, in two photocatalytic reactions of RhB solution and TC-HCl solution under visible-light irradiation, g-C_3_N_4_-mNb_2_O_5_ composites showed enhanced photocatalytic activity than mNb_2_O_5_. The 4% g-C_3_N_4_-mNb_2_O_5_ sample shows the highest photocatalytic activity. The other photocatalytic performance of Nb_2_O_5_/g-C_3_N_4_ composites were also tested by Ribeiro et al., Shi et al., and Li et al. [29,30,31]. Compared to the reported work, our work focus on the stability of catalysts, which is greatly distinct. The strategy based on the in situ hydrolysis can efficiently avoid the aggregation of Nb_2_O_5_, thus further facilitating the full exertion of catalyst activity. Besides, the interlayer of g-C_3_N_4_ sheets can be enlarged by the existence of Nb_2_O_5_, increasing the surface area of catalyst. The stability of our catalyst is superior to many reported literatures when evaluated in the photocatalytic of RhB and TC-HCl, which can be attributed to highly uniformity and tightly anchoring of Nb_2_O_5_ on g-C_3_N_4_ sheets. 

## 4. Conclusions

In summary, we have prepared a series of g-C_3_N_4_-mNb_2_O_5_ composites by in situ NbCl_5_ hydrolysis, deposition and solvothermal treatment. It is found that the g-C_3_N_4_-mNb_2_O_5_ composites exhibit lower PL intensity than pure mNb_2_O_5_ and g-C_3_N_4_ and narrower band gap than pure mNb_2_O_5_, leading to a significant enhancement of photocatalytic activity with excellent stability and recyclability for degradation of RhB and TC-HCl. Among the composites, 4% g-C_3_N_4_-mNb_2_O_5_ sample shows the highest photocatalytic activity under visible-light irradiation (*λ* > 420 nm), which is ascribed to the strong interaction between g-C_3_N_4_ and Nb_2_O_5_, its smallest band gap and lowest PL intensity. Based on the above characterization and photocatalytic results, the excellent photocatalytic activity of 4% g-C_3_N_4_-mNb_2_O_5_ can be attributed to combined effects between g-C_3_N_4_ and mNb_2_O_5_ as follows: (1) the induction of g-C_3_N_4_ into mNb_2_O_5_ leads to extension the absorption into visible light region; (2) the recombination of photogenerated electrons and holes is inhibited to some extent; (3) its highest surface area among all composites leads to more active sites; and (4) interaction exists between mNb_2_O_5_ and g-C_3_N_4_, as observed in XPS and FT-IR. These also account for the remarkable stability and recyclability of the catalyst 4% g-C_3_N_4_-mNb_2_O_5_ in the degradation reaction.

## Highlights

1. g-C_3_N_4_-mNb_2_O_5_ composites were obtained by in situ hydrolysis deposition and solvothermal treatment.
2. Under the visible light irradiation, 4% g-C_3_N_4_-mNb_2_O_5_ exhibited the highest photocatalytic activities to degrade rhodamine B (97.5% degradation within 180 min) and tetracycline hydrochloride (75.7% degradation within 60 min) solution with excellent stability and recyclability.
3. The g-C_3_N_4_-mNb_2_O_5_ composites showed narrower band gap, lower PL intensity and larger surface area than pure mNb_2_O_5_ and g-C_3_N_4_, leading to a significant enhancement of their photocatalytic activity.

## Supplementary Materials

The following are available online at http://www.mdpi.com/2079-4991/8/6/427/s1, Figure S1: A schematic diagram of photocatalytic equipment, Figure S2: Emission spectrum of Xe lamp with 420 nm filter, Figure S3: Influence of adsorption process on: (a) RhB solution (15 mg/L, 70 mL); and (b) TC-HCl solution (40 mg/L, 100 mL) containing 30 mg 4% g-C_3_N_4_-mNb_2_O_5_ powder under dark, Figure S4: Absorption changes of: (a) RhB solution (15 mg/L, 70 mL); and (b) TC-HCl solution (40 mg/L, 100 mL) containing 30 mg 4% g-C_3_N_4_-mNb_2_O_5_ powder under visible light irradiation, Figure S5: Standard curves of absorbance intensity vs. concentration of: (a) RhB solution; and (b) TC-HCl solution, Table S1: The elements content of 4% g-C_3_N_4_-mNb_2_O_5_ by EDS analysis.
