# Supplementary material for: Construction of g-C3N4-mNb2O5 Composites with Enhanced Visible Light Photocatalytic Activity
Source: Nanomaterials (Basel). 2018 Jun 12;8(6):427. doi: 10.3390/nano8060427 (PMC6027328; doi:10.3390/nano8060427)
Supplement: Supplementary file 1 [file nanomaterials-08-00427-s001.pdf]

Supplementary Materials

Construction of g-C_3N_4-mNb_2O_5 Composites with Enhanced Visible Light Photocatalytic Activity

Meiyin Wang, Hui Wang, Yuanhang Ren, Cheng Wang, Zhewei Weng, Bin Yue * and Heyong He *

Table S1. The elements content of 4% g-C_3N_4-mNb_2O_5 by EDS analysis.

Element	Peak Area	Area Sigma	k Factor	Weight %	Weight% Sigma	Atomic %
C K	1607	113	2.208	43.54	2.20	72.06
N K	127	63	2.965	4.61	2.21	6.54
O K	313	41	1.810	6.95	0.90	8.64
Cu K	1896	71	1.366	31.79	1.56	9.95
Nb K	384	43	2.779	13.11	1.40	2.81
Totals				100.00		

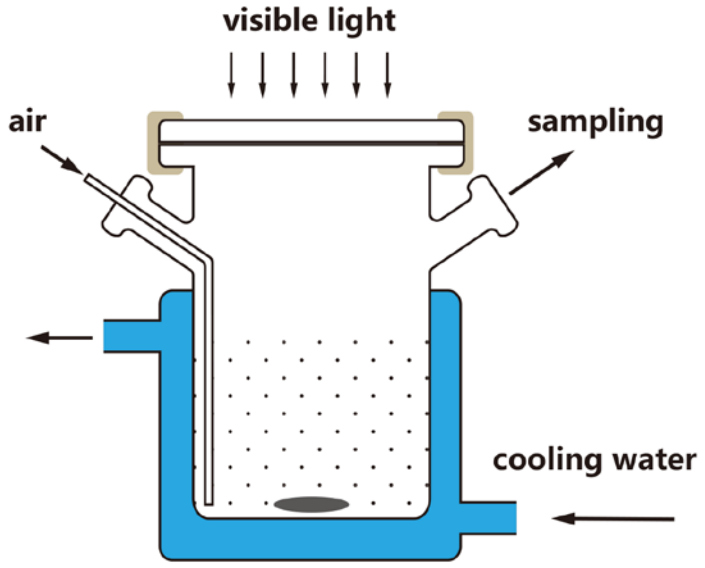

Figure S1. A schematic diagram of photocatalytic equipment.

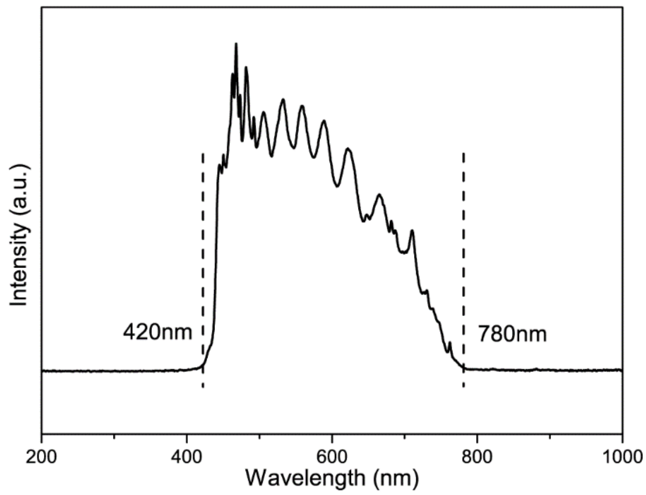

Figure S2. Emission spectrum of Xe lamp with 420 nm filter.

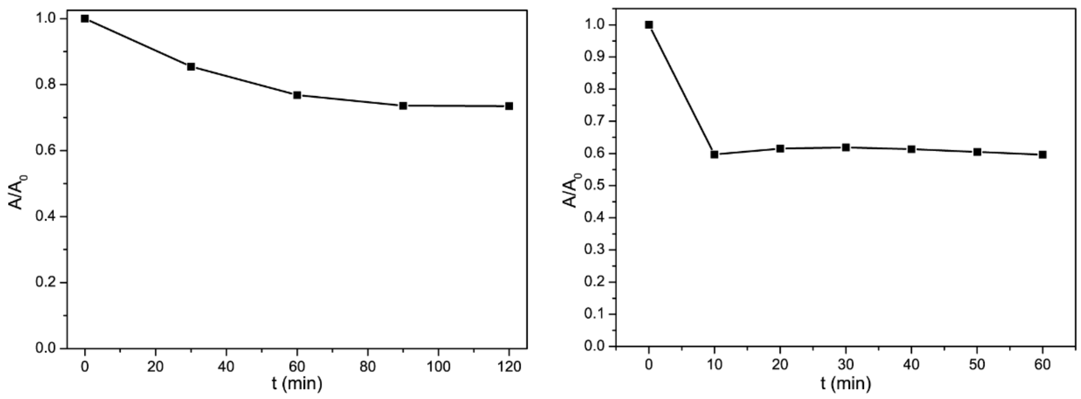

(**a**) (**b**)

Figure S3. Influence of adsorption process on: (**a**) RhB solution (15 mg/L, 70 mL); and (**b**) TC-HCl solution (40 mg/L, 100 mL) containing 30 mg 4% g-C_3N_4-mNb_2O_5 powder under dark.

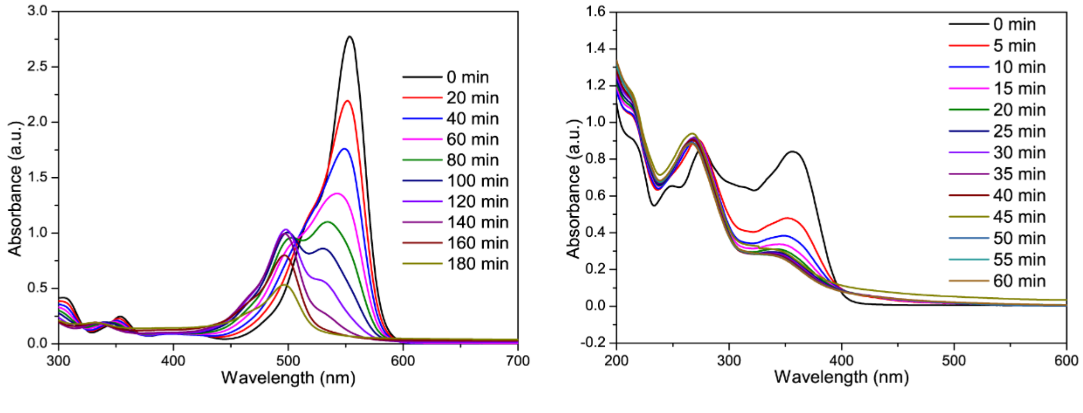

(**a**) (**b**)

Figure S4. Absorption changes of: (**a**) RhB solution (15 mg/L, 70 mL); and (**b**) TC-HCl solution (40 mg/L, 100 mL) containing 30 mg 4% g-C_3N_4-mNb_2O_5 powder under visible light irradiation.

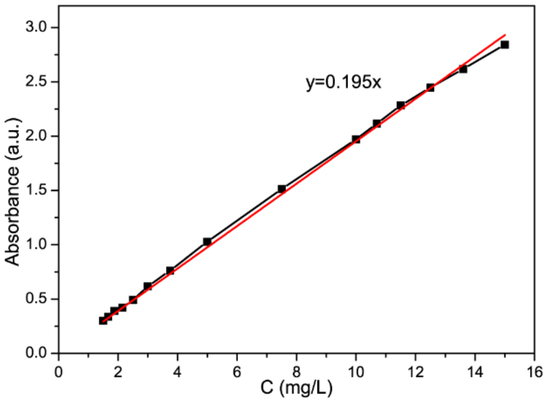

(**a**)

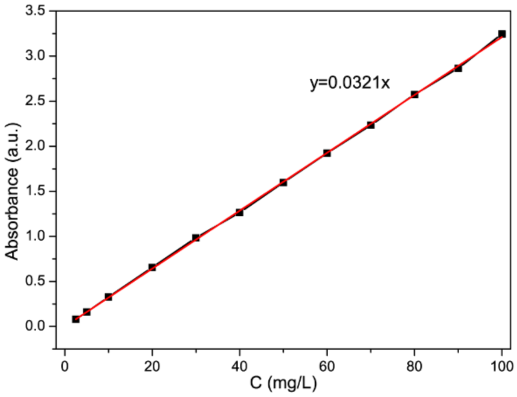

(**b**)

Figure S5. Standard curves of absorbance intensity vs. concentration of: (**a**) RhB solution; and (**b**) TC-HCl solution.
